# Electrochemical Creatinine (Bio)Sensors for Point-of-Care Diagnosis of Renal Malfunction and Chronic Kidney Disorders

**DOI:** 10.3390/diagnostics13101737

**Published:** 2023-05-13

**Authors:** Zohaib Saddique, Muhammad Faheem, Amir Habib, Iftikhar UlHasan, Adnan Mujahid, Adeel Afzal

**Affiliations:** 1 Sensors and Diagnostics Laboratory, School of Chemistry, University of the Punjab, Quaid-I-Azam Campus, Lahore 54590, Pakistan; 2 Key Laboratory of Polyoxometalate Science of Ministry of Education, Northeast Normal University, Changchun 130024, China; 3 Department of Chemistry, School of Science, University of Management and Technology, Lahore 54770, Pakistan; 4 Department of Physics, College of Science, University of Hafr Al Batin, P.O. Box 1803, Hafr Al Batin 39524, Saudi Arabia

**Keywords:** biosensors, creatinine, diagnostics, kidney failure, point-of-care

## Abstract

In the post-pandemic era, point-of-care (POC) diagnosis of diseases is an important research frontier. Modern portable electrochemical (bio)sensors enable the design of POC diagnostics for the identification of diseases and regular healthcare monitoring. Herein, we present a critical review of the electrochemical creatinine (bio)sensors. These sensors either make use of biological receptors such as enzymes or employ synthetic responsive materials, which provide a sensitive interface for creatinine-specific interactions. The characteristics of different receptors and electrochemical devices are discussed, along with their limitations. The major challenges in the development of affordable and deliverable creatinine diagnostics and the drawbacks of enzymatic and enzymeless electrochemical biosensors are elaborated, especially considering their analytical performance parameters. These revolutionary devices have potential biomedical applications ranging from early POC diagnosis of chronic kidney disease (CKD) and other kidney-related illnesses to routine monitoring of creatinine in elderly and at-risk humans.

## 1. Introduction

**Figure 1:**
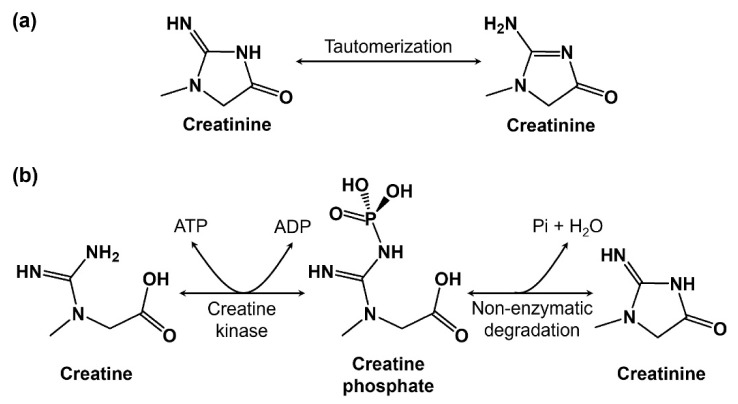
(**a**) A schematic representation of the molecular structure of creatinine: 2-amino-1-methyl-2-imidazoline-4-one. (**b**) The reversible enzymatic conversion of creatine-to-creatine phosphate by creatine kinase and the non-enzymatic production of creatinine from creatine phosphate by the removal of inorganic phosphate and a water molecule. (ATP: adenosine triphosphate; ADP: adenosine diphosphate; Pi: inorganic phosphate).

The clinical diagnosis of renal malfunction, acute kidney injury (AKI), or chronic kidney disease (CKD) is performed by monitoring creatinine concentration in human serum. Creatinine is a muscle’s metabolic waste, produced from the conversion of creatine and creatine phosphate with the release of energy [1,2], as shown in Figure 1. Creatinine enters the bloodstream, is filtered by the kidneys, and is excreted through urine [3]. Pottel et al. [4] and Ceriotte et al. [5] established normal creatinine levels in blood in separate studies (see Table 1). Creatinine levels above and below the normal range are considered toxic. Abnormal levels of creatinine may be a sign of diabetic nephropathy, eclampsia, glomerulonephritis, pre-eclampsia, pyelonephritis, a reduction in renal blood flow, renal failure, urinary tract obstruction, etc. Hence, creatinine is used as a disease biomarker in predicting important health outcomes.

In particular, serum creatinine levels are elevated due to loss of kidney function, and this is a clear indication of kidney disease [6]. Besides, creatinine is a significant biomolecule for monitoring post-surgery renal functions, which also stipulates the hydration level in the body [7,8]. Hence, it is important to monitor creatinine for timely diagnosis of kidney-related illnesses and to reduce the chances of renal failures, severe kidney disease, and death [9]. Various methods and materials have been developed for the recognition of creatinine in physiological fluids. Traditionally, the Jaffé method [10,11] is clinically used at centralized laboratories for creatinine diagnostics.

**Table 1:** Reporting the normal range and toxic level of creatinine in serum for different ages and genders [4,5,12].

Age (Years)	Gender	Normal Range (µM)	Toxic Level (µM)
<14	Male	22.11–73.39	<18, >80
	Female	22.11–73.39	<18, >80
15–20	Male	44.21–80.46	<35.37, >88
	Female	37.14–68.97	<31, >75.16
20–70	Male	61.89–106.1	<55, >113
	Female	44.21–88.42	<39, >95
>70	Male	>61.89–106.1	<45, >113
	Female	>44.21–88.42	<39, >95

However, in recent years, electrochemical (bio)sensors have become a reliable alternative for POC analysis due to their fast analytical response, high sensitivity and accuracy, and simplistic operation [13,14]. POC analyses require portable systems [15], and thanks to recent advancements, complex benchtop instruments can be replaced by miniaturized electroanalytical devices as small as a smartphone, permitting people to perform tests without extensive training. The electrochemical (bio)sensors, as portable POC disease diagnostics, either make use of biological receptors such as enzymes or employ synthetic responsive materials, which provide a sensitive interface for the creatinine-specific interactions. Herein, we present an overview of the characteristics of different receptors and electrochemical principles, along with their performance, limitations, and challenges.

## 2. Creatinine Receptors

### 2.1. Enzymatic Receptors

Biosensors are generally based on bioreceptors such as enzymes, DNA, etc. for the identification of molecular analytes and/or microorganisms [16,17,18,19,20]. Enzymes are catalytic proteins used to enhance the rate of a reaction without being consumed or changing the balance of chemical reactions. Enzymes use a lock-and-key model that affects a specific substrate or molecule, such as creatinine [21]. Enzymes convert creatinine into measurable substances, e.g., NH_4_^+^ and H_2_O_2_, which are detected via pH- and oxygen-sensitive electrodes to measure serum creatinine concentrations [9].

The attachment of an enzyme to an inert surface or solid support forming a complex matrix is termed immobilization, which is range-determining and affects the stability of sensors. Several immobilization techniques have been studied to develop sensors at the commercial level. For instance, Do et al. [22] performed the immobilization of creatinine deiminase (CD) enzyme by drop-casting glutaraldehyde, CD, and bovine serum albumin (BSA) solutions in a series on Nafion^®^-nsPANi/Au/Al_2_O_3_ electrodes to construct an amperometric creatinine biosensor. Other techniques include gel entrapment [23], crosslinking [24], polymer entrapment [25], and non-immobilized deposition [26]. Crosslinking offers greater stability but with reduced sensitivity and analytical range [27].

**Figure 2:**
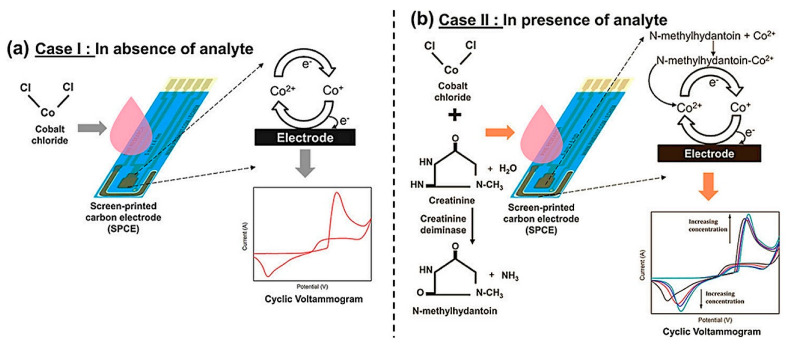
Principle of the electrochemical recognition of 1-methylhydantoin produced by enzymatic hydrolysis of creatinine and detected by complexation with Co^2+^ ions to produce a redox signal: (**a**) in the absence of the analyte, and (**b**) in the presence of the analyte. Reprinted from Dasgupta et al. [28], American Chemical Society (2020).

Dasgupta et al. [28] developed a single-enzyme-based amperometric sensor for the detection of creatinine that employed the enzymatic hydrolysis of creatinine to 1-methylhydantoin by creatinine deiminase. 1-methylhydantoin was subsequently detected by its complexation with the Co^2+^ ions to produce the redox signal, as shown in Figure 2. The enzymatic sensors exhibit excellent specificity in complex mixtures but are costly, have production and purification problems, and have stability issues [29,30].

### 2.2. Non-Enzymatic Receptors

Enzymeless or non-enzymatic receptors are synthetically sensitive materials based on polymers and nanostructures. To generate specificity toward creatinine and reduce the cross-sensitivity of polymeric receptors to other interfering substances, molecular imprinting is often employed. Molecular imprinting generates template-specific cavities in polymeric matrices for biomimetic selectivity and high affinity toward the targeted analyte [31,32,33]. MIPs are built with a mixture of monomers, crosslinkers, and non-reactive templates. Imprinting is considered a viable approach to mimicking natural recognition mechanisms like antibodies [34]. The selection of monomers and crosslinkers is very important, while the complete removal of the template is challenging [33,35]. Removal of the template generates creatinine-specific cavities, offering selective binding and recognition of creatinine in samples. MIPs are highly sensitive, selective, and stable for a long time compared to biological receptors [36].

**Figure 3:**
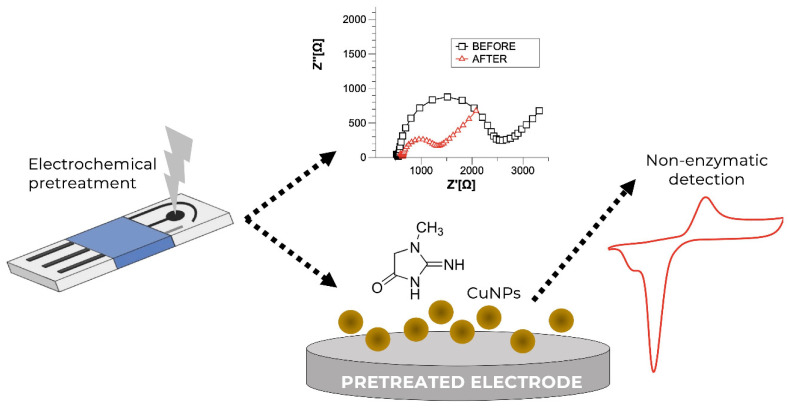
A schematic representation of a non-enzymatic creatinine sensor: A bare pre-treated screen-printed carbon electrode (PTSPCE) and electrodeposition of Cu nanoparticles (CuNPs); electrochemical impedance spectra of PTSPCE before and after CuNPs electrodeposition; and a cyclic voltammogram showing the non-enzymatic detection of creatinine. Reprinted from Domínguez-Aragón et al. [41], MDPI (Basel, Switzerland) (2023).

Likewise, novel physiochemical properties and adjustable size/shape make nanomaterials useful for different applications [37,38]. What makes them a possible interface for biosensors is their large surface-to-volume ratio, high absorption capacity, and stability [39]. Khusroshahi et al. [40] outlined the role of nanomaterials in improving the detection of early-stage CKD. Figure 3 shows an example of an electrochemical creatinine sensor using pre-treated screen-printed carbon electrodes (PTSPCE) with electrodeposited Cu nanoparticles (CuNPs). Domínguez-Aragón et al. [41] demonstrated that the non-enzymatic PTSPCE/CuNPs sensor can detect creatinine in a linear working range of 10–160 μM with a sensitivity of 0.2582 μA·μM^−1^ and a detection limit (LOD) of 0.1 μM. Nanomaterials improve the electron transfer between receptor and electrode, thereby enhancing the electrochemical signal [42]. Furthermore, nanocomposite materials made of (imprinted) polymers and different types of nanostructures have been designed and used as enzymeless receptors for electrochemical creatinine sensors.

## 3. Electrochemical Creatinine (Bio)Sensors

**Figure 4:**
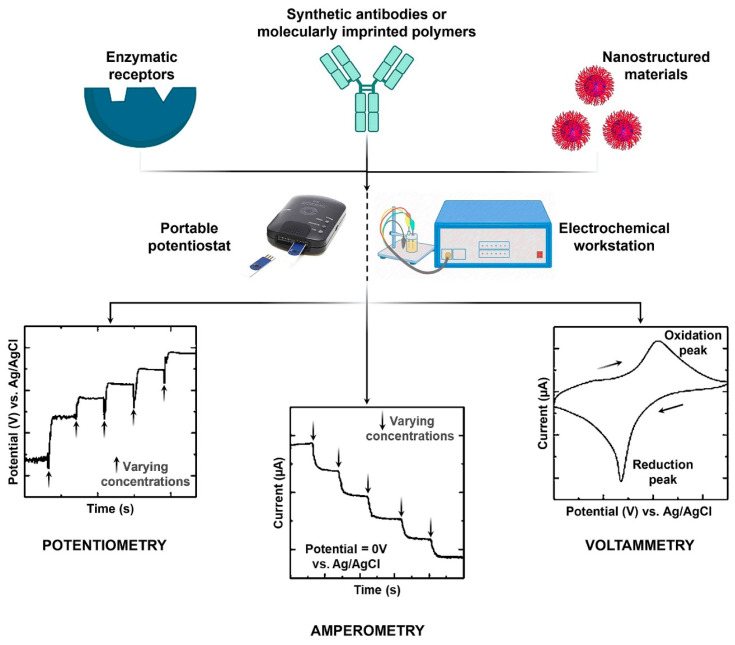
The principle of different electrochemical (bio)sensors: A combination of receptors, working electrodes, electrochemical detectors, and methods employed for creatinine diagnosis.

Electrochemical creatinine (bio)sensors are a viable alternative to conventional analytical methods and clinical approaches [43]. They are superior diagnostic devices due to their miniaturization capacity, fast response/analysis time, cost efficiency, deliverability, and range of techniques that enable sensitive detection of any biomarker. Figure 4 shows different types of electrochemical sensors used for the detection of creatinine. In an electrochemical method, creatinine is either directly measured using an electrochemical redox probe or converted into a measurable substance such as H_2_O_2_ or NH_4_^+^ for quantification [44,45].

The latter is usually performed by enzymatic receptors such as creatinine deiminase (CD), while in non-enzymatic methods, nanomaterials/polymers are used for the direct determination of creatinine using a redox probe such as [Fe(CN)_6_]^3−^/[Fe(CN)_6_]^4−^. The range of techniques used for the transduction of electrochemical signals is an added advantage [46]. These methods include cyclic voltammetry, amperometry, potentiometry, conductometry, etc. [47].

### 3.1. Amperometric Creatinine Sensors

In electrochemical reactions, the oxidation-reduction of chemical species results in electron flow, which is measured by amperometry. The basic setup of amperometric diagnostics employs a three-electrode system with working (WE), reference (RE), and counter (CE) electrodes [48]. Pt-wire is mostly used as CE [49,50], while other materials include Pt/Ag [51], glassy carbon electrode (GCE) [52], carbon rod/paste [53], graphite [54], and MnO_2_ [55]. On the other hand, Ag/AgCl is widely used as RE [52,56,57], while the application of saturated calomel electrodes (SCE) is also reported [53].

For WE, two enzymatic variants based on single-enzyme [54,58,59] or three-enzyme [48,57,60] systems have been used as receptors. They use CD, creatinine amidohydrolase, creatine amidinohydrolase, sarcosine oxide, and their derivatives. A single-enzyme system performs a one-step reaction with creatinine, producing N-methylhydantoin and ammonia, where the latter is measured [61]. Three-enzyme systems employ a step-wise reaction to produce glycine, formaldehyde, and H_2_O_2_ [57], as shown in Equations (1)–(3).

(1) $$\mathrm{creatinine}+H_{2}O\overset{\mathrm{creatinine}\;\mathrm{amidohydrolase}}{\to }\mathrm{creatine}$$

(2) $$\mathrm{creatine}+H_{2}O\overset{\mathrm{creatine}\;\mathrm{amidinohydrolase}}{\to }\mathrm{sarcosine}+\mathrm{urea}$$

(3) $$\mathrm{sarcosine}+H_{2}O+O_{2}\overset{\mathrm{sarcosine}\;\mathrm{oxidase}}{\to }\mathrm{glycine}+\mathrm{formaldehyde}+H_{2}O_{2}$$

Enzymes are generally immobilized onto the sensor surface using entrapment, cross-linking, or covalent methods. The immobilization can be facilitated by chemical agents such as carbodiimine [51], piranha mixture [62], glutaraldehyde [60], and glycerol or lactitol [58]. However, enzyme immobilization is often critical because it greatly influences the operational stability and response of the device. Nonetheless, enzymes offer unmatched selectivity toward creatinine.

Wei et al. [63] developed an amperometric creatinine biosensor based on antibody-based affinity interactions for selective recognition of creatinine in clinical serum samples. They used horseradish peroxidase (HRP)-conjugated anti-creatinine antibodies immobilized on a conducting polymer, i.e., polypyrrole, to detect creatinine. Besides, many non-enzymatic receptors have been developed for amperometric creatinine sensors. For instance, Nontawong et al. [64] synthesized CuO nanoparticles coated with a MIP shell (CuO@MIP) to fabricate a creatinine sensor. The sensor exhibited repeatable signals at different creatinine concentrations (0.5–200 µM) and good sensitivity. 

**Figure 5:**
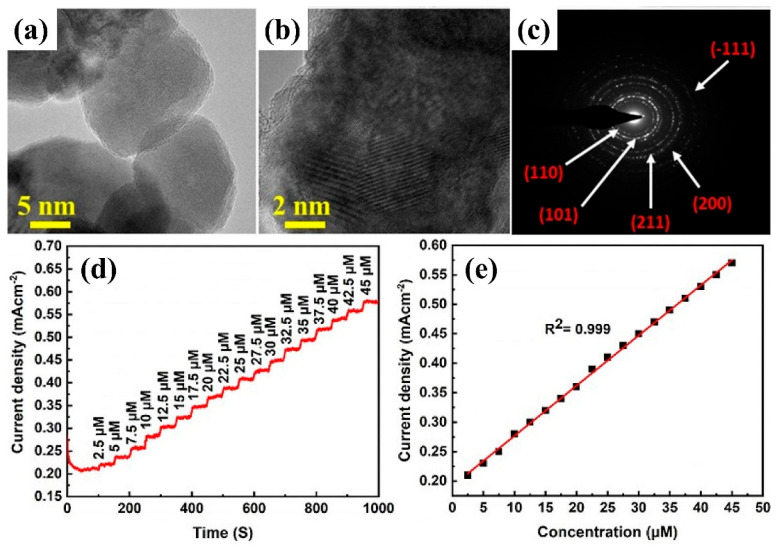
(**a**,**b**) HRTEM (high-resolution transmission electron microscopy) images of SnO_2_@Cu_2_O hybrid electrodes; (**c**) SAED (selected area electron diffraction) pattern of SnO_2_@Cu_2_O hybrid nanostructures; (**d**) amperometric sensor response of SnO_2_@Cu_2_O nanostructures toward different concentrations of creatinine; and (**e**) the analytical curve between current density and creatinine concentrations for calculating sensitivity. Adapted from Ullah et al. [65], American Chemical Society (2022).

Among the non-enzymatic receptors, Ullah et al. [65] reported the development of an amperometric creatinine sensor based on nanoporous anodic SnO_2_ decorated with Cu_2_O nanoparticles (see Figure 5). These SnO_2_@Cu_2_O hybrid nanostructures revealed ultra-high sensitivity (24343 µA/cm^2^.mM) toward creatinine with a broad linear detection range (2.5–45 µM) and a very low detection limit (2.3 nM). Furthermore, they tested the cross-sensitivity of the SnO_2_@Cu_2_O hybrid sensor toward a wide range of potentially interfering analytes, including ascorbic acid, cholesterol, L-cysteine, dopamine, glucose, urea, uric acid, etc. The sensor exhibited remarkable selectivity for creatinine detection.

### 3.2. Potentiometric Creatinine Sensors

Potentiometric (bio)sensors measure the potential difference between WE and RE in an electrochemical cell when zero or non-significant electric current is flowing between them [66]. The potential of WE changes with a change in the analyte concentration [67]. Many potentiometric creatinine biosensors reported so far are based on enzymes that catalyze the hydrolysis of creatinine, and detect pH by measuring liberated protons (H^+^ ions) [68] or ammonium (NH_4_^+^ ions) [29] resulting from creatinine hydrolysis. Potentiometric detection usually employs a two-electrode setup with an ion-selective electrode (ISE) and RE. ISEs mostly comprise Au [69], Pt [68,70], IrO_x_ [71], Teflon cylinder [72], graphite [73], Hg-drop electrode [74], or GCE [44], while Ag/AgCl acts as RE [44,69].

Meyerhof and Rechnitz [75] introduced the first potentiometric creatinine sensor with an ammonia-sensitive electrode. This ammonia gas was generated by creatinine hydrolysis using CD, or creatinine iminohydrolase [75], as shown in Equation (4).

(4) $$\mathrm{creatinine}+H_{2}O\overset{\mathrm{enzyme}}{\to }\mathrm{methylhydantoin}+{\mathrm{NH}}_{4}^{+}$$

Three-enzyme systems have also been employed in potentiometric creatinine detection. The enzymes CD, creatinine amidohydrolase, and urease were reportedly used for creatinine and urea sensing [29]. Urea produced from the hydrolysis of creatinine (Equations (1) and (2)) is further hydrolyzed by the urease to form NH_3_ and CO_2_, as shown in Equation (5). However, three-enzyme systems further reduce the operational stability of creatinine diagnostics, and layers of immobilized enzymes result in a significant loss of sensitivity.

(5) $$\mathrm{urea}+2H_{2}O\overset{\mathrm{urease}}{\to }{\mathrm{CO}}_{2}+{\mathrm{NH}}_{3}$$

The operational/storage stability of enzymatic receptors is the main problem hampering their use in practical applications. Maximum storage stability of >6 months at 4 °C [76] and operational stability of ~90 days with a 37% loss in sensitivity [77] have been reported.

**Figure 6:**
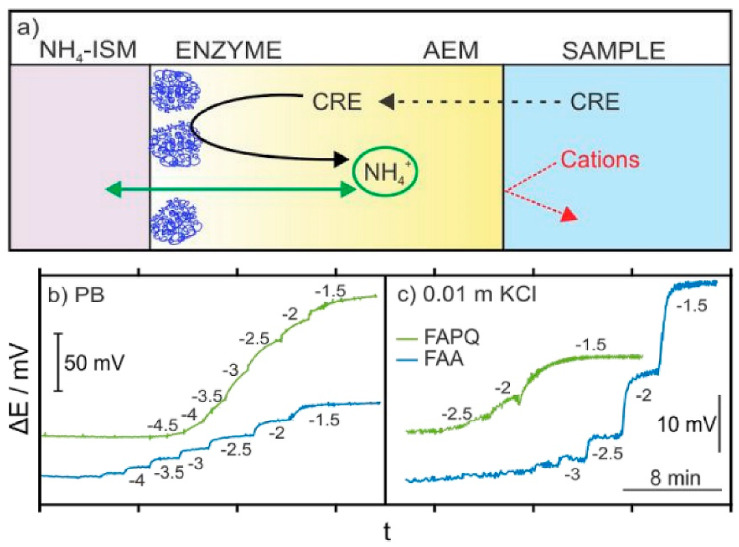
(**a**) Working mechanism underlying the all-solid-state creatinine biosensors. The responses of the electrodes prepared with FAPQ (NMP) and FAA (n-propanol) at 4 wt.% are displayed for increasing creatinine concentrations in (**b**) PB and (**c**) 0.01 M KCl backgrounds. NH_4_^+^SM—ammonium-selective membrane; AEM—anion-exchange membrane; PB—phosphate buffer; FU—Fumion-based membranes. The numbers in the plots indicate the logarithmic concentrations of creatinine. Adapted from Liu et al. [78], American Chemical Society (2020).

The interference from endogenous NH_4_^+^ ions in potentiometric creatinine detection is also a challenge. Liu et al. [78] recently proposed the use of an anion exchange membrane (AEM) as a barrier for charged interferences in the potentiometric determination of creatinine in non-diluted urine samples. As shown in Figure 6, creatinine diffuses through AEM and interacts with the creatinine deiminase to form NH_4_^+^ ions that are dynamically determined by the ammonium-selective electrodes (NH_4_^+^-ISM). Thus, possible interference from cationic species is avoided. The potentiometric creatinine biosensor exhibits excellent characteristics at 1–50 mM creatinine concentration. However, the response time is compromised (15–60 min) primarily because of slow creatinine diffusion across the AE.

Furthermore, to reduce such interference, ion-selective field-effect transistors (ISFET) are constructed with a thin layer of enzymes immobilized over the ion-selective membrane, thereby making them enzyme-sensitive FETs. ISFETs can be based on NH_4_^+^-selective FETs or pH-sensitive FETs [79,80]. Operational/storage stability, sensitivity, and potential inhibition to interference are determined by the design of the electrode surface and enzyme immobilization technique. 

Although most of these biosensors were based on enzymes, a few works focused on the development of enzyme-free potentiometric analysis of creatinine [66,81,82,83,84]. Guinovart et al. [83], for instance, prepared a novel ionophore based on calix [4]pyrrole for the detection of creatinine cations. Certain non-enzymatic sensors exhibit excellent characteristics, but they do not necessarily outperform enzymatic receptors.

### 3.3. Voltammetric Creatinine Sensors

In voltammetry, the current response of an analyte under an applied potential difference is measured and can be used to detect creatinine. For instance, Saidi et al. [85] developed a voltammetric method to measure urinary creatinine. They employed seven different electrodes alternatively as WE, including GCE, Ni, Pd, Pt, Cu, Ag, and Au, along with an Ag/AgCl RE. The voltammetric creatinine sensor exhibited excellent results that were comparable to Jaffe’s method. Although the voltammetric sensor offered the direct determination of creatinine without significant sample modification or pre-treatment, its portability, response range, and detection limits (LOD) were not comparable with modern analytical tools, rendering it clinically irrelevant. 

Gao et al. [86] developed a new creatinine sensing platform by modifying GCE with electrodeposited Cu nanoparticles on the PDA-rGO-NB layer. They used square-wave voltammetry to detect creatinine in a phosphate buffer solution (PBS). The sensor exhibited high sensitivity, a linear response range (0.01–100 µM), and a LOD of 2 nM. In a similar method, gold electrodes modified by Nafion mixed with graphene quantum dots in the dispersion of Cu^2+^ were used to prepare a voltammetric sensor for the detection of creatinine in urine samples [87]. This sensor offered a dynamic response range of 0.11–50.9 mg/L; however, its selectivity was compromised. To improve the sensitivity and selectivity of the voltammetric sensors, Sriramprabha et al. [88] developed Fe_2_O_3_/polyaniline nanocomposites for the detection of creatinine in serum. They reported a response range of 0.001–13 mM and a threshold LOD of 144 nM with enhanced sensitivity. The sensor did not need a bioreceptor or binder for the functioning or any pre-treatment of the sample.

**Figure 7:**
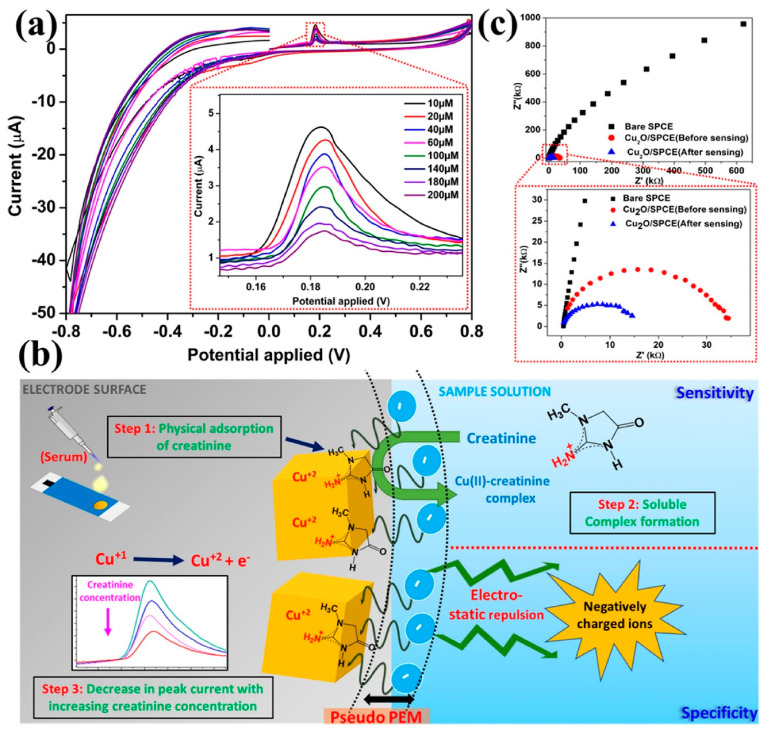
(**a**) Cyclic voltammetric response of SB3C16@Cu_2_O/SPCE to 10–200 μM creatinine, showing an inversely proportional relationship between the oxidative peak current and creatinine concentration. (**b**) Schematic of the proposed sensing mechanism, depicting the specificity imparted by the pseudo-PEM and sensitivity due to the potential-assisted soluble Cu(II)-creatinine complex formation. (**c**) The Nyquist plot confirms the decrease in the *R_ct_* value of the Cu_2_O nanoparticle-modified SPCE post-creatinine quantification, indicating an increase in the overall electrode conductivity. SB3C16: *N*-hexadecyl-*N*,*N*-dimethyl-3-ammonio-1-propanesulfonate; SPCE: screen-printed carbon electrodes. Reprinted from Kumar et al. [89], American Chemical Society (2023).

Recently, Kumar et al. [89] utilized zwitterion (*N*-hexadecyl-*N*,*N*-dimethyl-3-ammonio-1-propanesulfonate) functional Cu_2_O nanoparticles to generate a pseudo-proton-exchange membrane that was believed to electrostatically hinder interfering substances from reaching the surface of electrodes, as shown in Figure 7. Hence, the specificity of the voltammetric creatinine sensor was enhanced using an enzymeless approach without a significant loss in sensitivity. The creatinine sensor demonstrated high specificity against different interfering analytes such as acetic acid, glucose, ascorbic acid, urea, and uric acid. They also observed a linear response to 10–200 μM creatinine concentrations with a fast response time (<50 s) and excellent reproducibility.

### 3.4. Other Electrochemical Sensors

The versatility of electrochemical techniques allows the determination of creatinine using different principles. In addition to the abovementioned electrochemical diagnostics, researchers also made use of electrochemical impedance spectroscopy (EIS), conductometry, and capacitive/dielectric measurements to monitor creatinine in PBS and physiological fluids. For instance, Reddy and Gobi [90] developed an impedimetric sensor using a molecularly imprinted poly(methacrylic acid) copolymer. The charge-transfer impedance (*R_ct_*) of the MIP sensor was monitored at different concentrations of creatinine. The MIP impedimetric sensor exhibited a low detection limit (~20 ng mL^−1^) and excellent selectivity in the presence of possible interferents.

Isildak et al. [91] developed a highly sensitive and stable conductometric sensor. They immobilized creatininase as a receptor on an NH_4_^+^-sensitive membrane using covalent immobilization and measured liberated NH_4_^+^ ions. The sensor had a fast response time (10 s) and an acceptable detection limit/range, but only 4 weeks of operational/storage stability [91]. On the other side, Breaik et al. [92] immobilized creatinine deaminase as a receptor entrapped on the surface of poly(vinyl alcohol), polyethyleneimine, and Au nanoparticle composite films. The sensor exhibited a LOD (2 µM), a linear detection range (100–600 µM), and a response time of 3 min. Operational/storage stability was not reported, which is the major concern of enzymatic sensors. Conversely, the advantages of the conductometric sensor include the absence of RE, high sensitivity, good compatibility, low cost, and miniaturization capacity.

Capacitive sensors employed MIPs as dielectrics, and the dielectric constant and storage capacity changed when creatinine diffused into MIP [93]. The first capacitive creatinine was based on an artificial chemoreceptor, i.e., MIP [94]. It was a reversible chemosensor with a LOD of 10 µM. Since capacitive sensors do not involve any reaction, pH does not influence response [93]. The thickness of MIP, however, is the key factor, as an increase in film thickness causes a 10% change in the sensor signal. Guha et al. [95] constructed a label-free capacitive sensor using a complementary metal oxide near-field dielectric at 6 GHz. The sensor offered a suitable detection range of 0.88–880 µM and required only 2 µL of the sample. Its reduced size and low fabrication cost made it suitable for commercial use as a portable device.

## 4. Limitations and Challenges

Table 2 provides a comparison of the experimental conditions and analytical performance of enzymatic and non-enzymatic electrochemical creatinine (bio)sensors. Typically, electrochemical diagnostics offer unparalleled features such as high sensitivity, a wide detection range, cost efficiency, and deliverability [96]. The small size, portability, and possibility to further miniaturize these devices are their greatest advantages in terms of accomplishing POC diagnostic applications. They require small sample volumes and minimal sample pre-treatment for the analysis and determination of creatinine. When compared with conventional analytical methods, one of the most significant features of these devices is their response time, which is often less than 1 min in the majority of reported studies [58,97,98,99] with 2–5 s also reported [42,53,100,101]. Another advantage of electrochemical diagnostics is their low LOD, which is often lower than normal creatinine levels in serum, saliva, and urine, e.g., 1.5 × 10^−11^ M [102].

On the contrary, electrochemical diagnostics must deal with some inherent limitations compared to conventional analytical methods. Firstly, they are less selective and often falter in complex solutions or physiological fluids. Enzymatic receptors are perceptibly selective toward creatinine and its hydrolytic products and yield excellent specificity [76,101]. However, the matrix for enzyme immobilization and electrode sensitivity to these products need to be optimized for superior performance. Enzyme immobilization is a great challenge, while the major drawback of enzymatic receptors is their operational/storage stability, which is usually <1 month with a significant loss in sensitivity [99,103]. The maximum operational stability reported so far for the amperometric biosensors is ~6 months with a 4–15% loss in activity [42,101,104], while potentiometric biosensors exhibit ~1-year operational stability with a 30–43% loss in sensitivity [105,106]. Similarly, the storage stability of enzymatic receptors does not match the practical requirements.

Non-enzymatic receptors based on MIPs/nanomaterials are advantageous in terms of operational/storage stability and competitive in terms of sensitivity/LOD. However, their stability is hardly reported in the literature. Kalaivani et al. [107] recorded ~8 months of operational stability with a 10% loss in sensitivity of a voltammetric sensor coated with inulin-based MWCNT-TiO_2_ receptors. The greater challenge of enzymeless sensors, however, is compromised selectivity, which must be substantially improved for clinical applications. For this purpose, molecular imprinting and intrinsic affinity characteristics of functional polymers/nanomaterials can be utilized and optimized [83,87,102]. Overall, enzymeless receptors provide an excellent alternative due to their cost-efficiency and straightforward processing, reduced number of steps involved in fabrication, competitive sensitivity, greater stability, and more freedom in the choice and optimization of receptor material.

The electrochemical (bio)sensors do not yet meet the criteria for deliverable POC creatinine diagnostics. A deliverable device must be compact in design, economical, robust, stable, sensitive, specific, and reproducible. Although the electrochemical devices attained many of these characteristics simultaneously, they often lacked one or another area. The pertinent literature lacks cohesive and comprehensive studies aiming at addressing these criteria in a series of experiments. Hence, a systematic approach that interconnects and tackles the prerequisites of a deliverable POC device for creatinine diagnosis is needed. Nonetheless, these electrochemical sensors are expected to be the first in the race for modern creatinine diagnostics for POC applications, and this realization could be a breakthrough in remote CKD diagnosis.

**Table 2:** A comparison of the composition, conditions, parameters, and analytical performance of the reported electrochemical creatinine (bio)sensors.

Material or Matrix	Enzymes ^1^	Electrodes ^2^		Range ^3^ (µM)	LOD ^4^ (µM)	τ_res_^5^ (s)	Sensitivity ^6^ (mA/unit.M)	Stability in Days ^7^ (Loss in Activity)	Recovery ^8^ (%)	Ref.
		WE	RE							
ZnO/chitosan/carboxylated MWCNT/polyaniline	CA, CI, SO	Pt	Ag/AgCl	10–650	0.5	10	0.030 μA/μM·cm^2^	120 (−15%)	98.7	[42]
Carboxylated MWCNT/polyaniline	CA, CI, SO	Pt	Ag/AgCl	10–750	0.1	5	40 μA/μM·cm^2^	180 (−15%)	–	[42]
Polypyrrole	–	Au	Au	0–1001.8	40.7	<300	–	–	–	[63]
Fe_3_O_4_/chitosan-graft-polyaniline	CRN, CR, SO	Pt	Ag/AgCl	1–800	1	2	3.9 μA/μM·cm^2^	200 (−10%)	99.93	[101]
AuNPs, MWCNTs	CRN, CR, SO	Teflon cylinder	Ag/AgCl	3–1000	0.1	9	1.32 µA/mM	–	98	[99]
Copper-polyaniline nanocomposite	CD	Carbon	Ag	1–125	0.5	15	85 mA/M·cm^2^	3	–	[58]
Polymethylene blue	–	Cu–doped carbon	Ag/AgCl	2.2–132.6	2 × 10^−4^	–	0.133 μA/ng·mL	180 (−4%)	98.7	[104]
Nafion-nsPANI	CD	Au	Ag/AgCl	100–400	–	–	1300 μA/mM·cm^2^	–	–	[22]
CuO@MIP	–	CPE	Ag/AgCl	0.5–200	0.083	–	0.21 µA/µM	14 (−20%)	–	[64]
ABTS^+^/CNT	–	Carbon	Ag/AgCl	0–21300	11	60	27.3 µA/mM·cm^2^	–	–	[56]
Nafion/Polyaniline	CD	Au	Au	10–1000	2	–	–	30 (−20%)	–	[108]
Sb/NPC	–	GCE	Ag/AgCl	–	0.744	–	–	–	90	[52]
Prussian blue	CA, CI, SO	CGP	–	50–1400	–	180	–	120 (−14%)	–	[57]
Conductive layer	CD	NH_4_^+^ ISE	–	5–255	3	25	–	–	–	[98]
β-cyclodextrin/poly(3,4-ethylene dioxythiophene)	–	GCE	SCE	100–10,000	50	60	–	30 (−5%)	–	[81]
Fe_3_O_4_@polyaniline	–	GCE	–	0.02–1	0.18	–	–	30 (−10%)	104.9	[82]
PVA-styryl pyridinium	CD	pH–FET	Ag/AgCl	20–2000	20	120–180	40	<1	–	[103]
Silicalite	CD	pH–FET	Ag/AgCl	0–2000	5	–	40	365 (−43.3%)	–	[105]
BEA gold (Zeolites)	CD	pH–FET	Ag/AgCl	0–2000	10	–	40	–	–	[80]
Calix [4]pyrrole	–	GCE	Ag/AgCl	10–10,000	~1		54.1	–	–	[83]
Zeolite paraffin	–	CPE	Ag/AgCl	0.1–100	0.079	<50	52	–	–	[84]
MWCNTs	CD	CPE	Ag/AgCl	1000–50,000	–	900–3600	57.01	–	–	[78]
Cu_2_O@MIP	–	SPCE		0–0.075	0.022	–	2.16 µA/nM	35	93	[109]
Polyethyleneimine/phosphotungstic acid	–	ITO	SCE	0.125–62.5	0.06		200	–	–	[110]
Cu	–	Carbon	SCE	6–378	0.0746	–	–	–	98	[111]
rGO-AgNPs	–	GCE	–	5 × 10^−5^–1.5 × 10^–3^	1.51×10^−5^	–	–	–	100	[102]
2-hydroxymethacrylate/methyl methacrylate/graphene oxide	–	GCE	–	44.2–26.2	16.6	120	–	–	–	[112]
MWCNTs-inu-TiO_2_	–	CPE	Ag/AgCl	0.2–1000	0.06	–	–	240 (−10%)	–	[107]
Nafion/graphene QDs	–	Au	Ag/AgCl	0.97–450	–	–	–	–	–	[87]
Cobalt chloride	–	Carbon	AgCl	44–354	–	–	–	–	–	[28]
AgNPs/folic acid/MWCNTs	–	CPE	SCE	0.01–200	0.008	1.5	50	14 (−5%)	96	[53]

^1^ Enzymes used as bioreceptors for creatinine identification. CA—creatinine amidohydrolase; CD—creatinine deiminase; CI—creatine amidinohydrolase; CR—creatinase; CRN—creatininase; SO—sarcosine oxidase. ^2^ Electrodes—materials used as working electrodes (WE) or reference electrodes (RE). CGP—carbon-graphite paste electrode; COE—Clark-type oxygen electrode; CPE—carbon paste electrode; GCE—glassy carbon electrode; ISE—ion-selective electrode; SCE—saturated calomel electrode. ^3^ Range—linear detection range in µM. ^4^ LOD—limit of detection in µM. ^5^ τ_res_—response time in seconds. ^6^ Sensitivity of the sensors is calculated from the respective calibration curves in milliamperes per unit concentration unless otherwise specified. ^7^ The operational stability in the number of days along with the percent loss in the activity (response) of the sensors are given in parenthesis. ^8^ Recovery—the percent recovery of creatinine in real samples such as serum, urine, etc. spiked with a known concentration of the analyte.

## 5. Concluding Remarks

Creatinine is a metabolic waste product and a biomarker for renal malfunction and kidney-related illnesses. Hence, it is important to measure it precisely and accurately in different physiological fluids. Considering their compact and robust nature, high sensitivity, wide working range, rapid response, cost efficiency, and unparalleled portability, the electrochemical (bio)sensors meet the commercialization prerequisites for the medical determination of creatinine. However, present electrochemical diagnostics exhibit certain shortcomings. To overcome these challenges, especially in producing deliverable and reproducible electrochemical devices devoid of interferences, there is a need to revisit the development strategy; to produce economical and robust synthetic receptors that can selectively interact with creatinine in physiological fluids; to either employ enzymeless systems or optimize enzyme immobilization methods for enhanced storage and operational stability; and to avoid pre-treatment of biological samples for rapid screening and ease-of-use. Portable and compact detectors, such as smartphone-enabled devices, must be developed for remote analysis and diagnosis of creatinine. These developments are expected to revolutionize and commercialize electrochemical creatinine diagnostics for practical POC applications.

## Data Availability

The data presented in this study are contained within the article.
